# Articulatory–kinematic changes in speech following surgical treatment for oral or oropharyngeal cancer: A systematic review

**DOI:** 10.1111/1460-6984.13148

**Published:** 2024-12-18

**Authors:** Thomas B. Tienkamp, Teja Rebernik, Rachel A. D'Cruz, Rob J. J. H. van Son, Martijn Wieling, Max J. H. Witjes, Sebastiaan A. H. J. de Visscher, Defne Abur

**Affiliations:** ^1^ Centre for Language and Cognition Groningen University of Groningen Groningen the Netherlands; ^2^ Department of Oral and Maxillofacial Surgery University of Groningen, University Medical Centre Groningen Groningen the Netherlands; ^3^ Research School of Behavioral and Cognitive Neurosciences University of Groningen Groningen the Netherlands; ^4^ Netherlands Cancer Institute Amsterdam the Netherlands; ^5^ Amsterdam Centre for Language and Communication University of Amsterdam Amsterdam the Netherlands

**Keywords:** articulation, articulatory–kinematics, oral and oropharyngeal cancer, speech

## Abstract

**Background:**

Treatment for oral or oropharyngeal squamous cell carcinoma (O&OSCC) often leads to problems with speech articulation. Articulatory–kinematic data may be especially informative in designing new therapeutic approaches for individuals treated for these tumours.

**Aims:**

To provide a systematic review of the literature assessing the articulatory–kinematic consequences of oral and oropharyngeal cancer treatment.

**Methods & Procedures:**

Five databases (PubMed, Embase, Scopus, Web of Science and PsycInfo) were used to identify studies that used kinematic methods to characterize the speech of individuals treated for O&OSCC. Risk of bias was assessed using the critical appraisal checklist from the Joanna Briggs Institute. Data were synthesized using the Synthesis Without Meta‐Analysis guidelines.

**Outcomes & Results:**

In total, 29 studies with a total of 197 individuals treated for O&OSCC were included. In most studies the risk of bias was moderate to high and certainty of evidence was very low to low. Results showed both global changes (i.e., reduced movement and increased asymmetry of the tongue) as well as more local changes (i.e., reduced palatal contact and more centralized productions of consonants) following treatment for O&OSCC. Generally, reported changes were related to tumour size and location. Smaller tumours resulted in better or more typical articulatory–kinematic speech outcomes. Articulatory movements were most reduced in the affected region of the tongue as compared with neighbouring parts. Study findings were limited to small sample sizes with generally minimal descriptions of patient characteristics. No study assessed the influence of primary radiation treatment or adjuvant radiation therapy on kinematic speech outcomes.

**Conclusions & Implications:**

Based on the literature to date, surgical treatment for O&OSCC seems to reduce articulatory–kinematics of speech, and post‐treatment outcomes may be partially explained by tumour size and location. The absence of studies assessing the effect of primary or adjuvant radiation therapy on articulatory–kinematics limits our knowledge of how these interventions influence post‐treatment kinematic speech outcomes. Future studies should provide detailed patient descriptions and develop standardized speech assessment tools in order to further our knowledge regarding articulatory–kinematic speech changes following treatment, and to move towards the development of active rehabilitation strategies for those with O&OSCC.

**WHAT THIS PAPER ADDS:**

**What is already known on this subject**

- Treatment for O&OSCC can result in problems with speech articulation.
- Speech outcomes for O&OSCC are highly variable.

**What this paper adds to the existing knowledge**

- We systematically reviewed and synthesized the literature on articulatory–kinematic changes following O&OSCC treatment and identified 29 related studies.
- Treatment for O&OSCC resulted in global (i.e., more asymmetrical or reduced movement patterns) and local (i.e., reduced palatal contact) articulatory–kinematic changes.
- In general, smaller tumours resulted in better or more typical articulatory–kinematic speech outcomes as compared with larger tumours.
- Articulatory movements were most reduced in the affected region of the tongue as compared with neighbouring parts.

**What are the potential or actual clinical implications of this work?**

- Understanding articulatory–kinematic impacts of surgical intervention for O&OSCC can guide (the development of) tailored speech rehabilitation.

## INTRODUCTION

Tumours affecting the oral cavity or the oropharynx affect an estimated 476,100 people worldwide each year, comprising about 2.5% of all cancer incidences (Ferlay et al., 2021). Squamous cell carcinoma (SCC) is the most common type of oral cancer, accounting for nine out of 10 cases (Bagan et al., 2010). Once diagnosed, oral or oropharyngeal cancer treatment depends on several factors such as tumour size and location, the aetiology, and the preferences and clinical condition of the individuals themselves. Generally, treatment consists of either surgical resection or a (chemo)radiation‐based therapy (Constantinescu & Rieger, 2019). Treatment modalities may be combined, especially for larger tumours, as local recurrence remains high (Cohan et al., 2009).

Among all cancer types, treatment for oral or oropharyngeal squamous cell carcinoma (O&OSCC) has one of the highest risks of loss or damage to vital functions such as swallowing and speech (Kreeft et al., 2009). For surgical treatment, depending on the resection site, some of the side‐effects include problems with swallowing (dysphagia; Borggreven et al., 2007; de Vicente et al., 2021; Lam & Samman, 2013) or altered/loss of sensation in the oral cavity (Loewen et al., 2010). For radiation‐based treatments, some of the side‐effects include dysphagia (Lazarus, 2009; Logemann et al., 2008); altered taste (Hovan et al., 2010); dry mouth (xerostomia; Chi et al., 2015); mucositis (Maria et al., 2017); and an immobile jaw (trismus; Lee et al., 2015). For a more comprehensive overview of O&OSCC treatment side‐effects, see Prelec and Laronde (2014). These functional problems may affect daily activities such as eating. Moreover, these functional problems may contribute to a reduced quality of life post‐treatment (Dwivedi et al., 2012; Epstein et al., 1999; Mowry et al., 2006). Another functional issue that often arises in individuals treated for O&OSCC, are problems with speech. Tissue loss, tethering of the remaining tissue, and scar tissue as a result of surgery, or limited tongue mobility due to radiation side‐effects complicate articulation (Constantinescu & Rieger, 2019; Jacobi et al., 2013; Laaksonen et al., 2011). The resulting speech may be less intelligible, which complicates everyday communication, workplace reintegration, and could lead to social isolation and a reduced quality of life (Dwivedi et al., 2009; Epstein et al., 1999; Meyer et al., 2004). Unsurprisingly, individuals treated for O&OSCC rank speech in their top priorities post‐treatment (Rogers et al., 2002; Tschiesner et al., 2013). Therefore, an understanding of treatment induced changes in speech is paramount, as it can inform rehabilitation strategies and improve post‐treatment quality of life.

The speech of individuals treated for O&OSCC has been analysed in a number of studies by means of perceptual and acoustic methods. Perceptual evaluations found that important factors affecting the degree of reduced intelligibility are the size of the resection (with better intelligibility after smaller excisions; Bressmann et al., 2004; Nicoletti et al., 2004; Pauloski et al., 1998), tongue mobility (with better intelligibility if the tongue was more mobile post‐treatment; Bressmann et al., 2004; Matsui et al., 2007), and adjuvant radiation therapy (with better intelligibility if the individual did not receive adjuvant radiation therapy; Matsui et al., 2007). Acoustic studies have analysed the speech signal in more detail and found that individuals treated for O&OSCC experience the most problems with sibilants (/s, ʃ/; Acher et al., 2014; Jacobi et al., 2013; Laaksonen et al., 2011; Tienkamp et al., 2023; Zhou et al., 2013) and plosives (e.g., /t, k/; de Bruijn et al., 2009; Jacobi et al., 2013). Moreover, vowels produced by individuals treated for O&OSCC may be pronounced less distinctly (i.e., the vowel space area becomes smaller; de Bruijn et al., 2009; Jacobi et al., 2013; Takatsu et al., 2017).

Perception and acoustic studies have contributed greatly to the understanding of changes in the speech of individuals treated for O&OSCC, and their findings have already been systematically reviewed (Balaguer et al., 2020; Dwivedi et al., 2009; Lam & Samman, 2013); however, perceptual and acoustic investigations only provide indirect evidence of treatment induced articulatory–kinematic function (i.e., movements of speech articulators). In order to investigate the source of an individual's speech problem, the actual kinematics of the tongue, jaw, and lips need to be tracked directly. Given the quality‐of‐life impact of the treatment and the rated importance of speech by individuals treated for O&OSCC, a systematic review synthesizing articulatory–kinematic changes is important for two reasons. First, understanding how articulatory–kinematics are impaired may inform speech–language therapists in designing more effective rehabilitation strategies, as current standardized therapies are almost non‐existent (Bressmann, 2021). Second, a systematic review of articulatory–kinematic changes is informative for surgeons, too, as it could further inform reconstruction guidelines in order to optimize speech outcomes post‐surgery.

Therefore, the main aim of this systematic review was to evaluate to what extent treatment for O&OSCC affects the articulatory–kinematics of the tongue, jaw, and lips. The second aim was to evaluate to what extent articulatory–kinematics were related to the following clinical variables: the tumour‐node‐metastasis (TNM) staging of the tumour; tumour location; and the primary treatment modality. The third aim was to evaluate whether articulatory–kinematic changes were more severe for individuals treated for O&OSCC with adjuvant radiation therapy as compared with without. The fourth and final aim of our review was to evaluate how the time post‐treatment relates to the magnitude of possible articulatory–kinematic changes.

## METHODS

The systematic review was conducted using the Preferred Reporting Items for Systematic review and Meta‐Analyses Protocol (Moher et al., 2015; Shamseer et al., 2015). The review was pre‐registered at the International Prospective Register of Systematic Reviews (PROSPERO)^1^ under registration number CRD42022340489 on 28 June 2022. A full protocol is documented in Tienkamp et al. (2022).

### Information sources and search strategy

Five databases were systematically searched by the first author [TT]: PubMed, Embase, Scopus, Web of Science, and PsycInfo. The search was completed on 8 February 2023. No lower limit for the publication date was imposed since articles suitable for the review were expected to be scarce. Based on previous systematic reviews and consultation with a research librarian, relevant search terms were selected and these are summarized in Table 1. Each query component (e.g., population and disease) was linked using the Boolean operator ‘AND’. Our full query for each database can be found in the Supplementary Materials (see Table S1 in the Supporting Information section). The palate and velopharynx were not included in the queries, since we were interested in active articulators that would be impacted by oral and oropharyngeal cancer.

**TABLE 1:** Keywords used in the systematic search.

Query relating to …	Keyword
Population and disease	Oral squamous cell carcinoma, squamous cell carcinoma, Oral cancer, Oral tumo*, Oral carcinoma, Mouth cancer, Mouth tumo*, Mouth carcinoma, Oropharyngeal cancer, Oropharyngeal tumo*, Oropharyngeal carcinoma, Head and neck cancer, Head and neck tumo*, Head and neck carcinoma, Facial cancer, Facial tumo*, Facial carcinoma, Tongue cancer, Tongue tumo*, Tongue carcinoma, Glossectom*, Post‐glossectom*, Postglossectom*
Outcome	Movement, articulation, speech, intelligibility, acousti*, phoneti*, speech perception, speech therapy, tongue displacement, tongue motion, tongue positio*, lingual movement, lingual displacement, jaw displacement, tongue movement, jaw movement, lip displacement, lip movement, lip aperture, asymmetr*, symmetr*, concav*, tongue tip elevation
Method	Magnetic resonance imag*, MRI, rt‐MRI, rtMRI, Real‐time MRI, cine‐MRI, ultrasound, UTI, ultrasound tongue imaging, EMA, electromagnetic articulography, EPG, electropalatography, palatography, vocal tract, linguopalatal contact, Videofluoroscop*, x‐ray, x‐ray microbeam

### Eligibility criteria

A study was considered eligible if the study: (1) contained at least one adult (18+) that was treated for oral or oropharyngeal cancer. All treatment modalities (i.e., surgery, chemoradiation or radiation) were included; (2) included a description of the tumour and/or treatment details; (3) measured and analysed speech articulation using kinematic methods; (4) was subjected to peer review (including conference proceedings); and (5) was written in English, French, German or Dutch.

### Selection process and data collection

All records that were identified with our search string were imported into the Zotero reference manager and deduplicated using the ‘Duplicate Items’ tab. Next, the deduplicated records were imported into Rayyan for title and abstract screening (Ouzzani et al., 2016). Each record was screened by two investigators [TT and RD] using the eligibility criteria described in 2.2. Any uncertainty was resolved through discussion together with a third investigator [TR]. Records that passed the initial abstract and title screening were reviewed by two investigators [TT and RD]. Disagreements were resolved through discussion together with a third investigator [TR]. For both abstract and full‐text screening, investigators were blinded to each other's decisions.

The first author [TT] extracted the following information from all included articles: authors, year of publication, study design, language and location of the study, participant characteristics (e.g., sample size, age, sex), tumour and treatment characteristics (e.g., TNM classification, location, treatment modality, reconstructive details), experimental task used (e.g., stimuli), outcome measures, summary of the quantitative and qualitative results, and any description of individual data if present. The full data extraction form can be found in Tienkamp et al. (2022).

### Risk of bias and confidence in evidence assessment

Risk of bias assessment was performed by the first author [TT] for each study according to the methodological design (e.g., a cohort or case‐control study) using the critical appraisal list provided by the Joanna Briggs Institute (Munn et al., 2020). These checklists assess the methodological quality of an individual using yes/no questions on the following domains: chosen variables, chosen participant groups, outcome measurement (reliability), and statistical analysis. The score of each study had to be above 50% to be included in the synthesis. We interpreted scores of 50–65%, 66–80% and ≥ 80% as high, moderate and low risk of bias, respectively.

The quality of the body of evidence was assessed for each study using the GRADE guidelines (Grades of Recommendation, Assessment, Development and Evaluation) from the GRADE working group (Guyatt et al., 2008). Since our systematic review will most likely not include randomized controlled trials, the quality of the body of evidence will start at a low rating.

### Synthesis method

In line with our protocol, our systematic review employs a narrative synthesis of extracted group or individual data to summarize and explain the characteristics and outcomes of the included studies in relation to the research aims specified in the introduction. Outcomes are reported using the Synthesis Without Meta‐Analysis (SWiM) guidelines (Campbell et al., 2020).

## RESULTS

### Study selection

**FIGURE 1:**
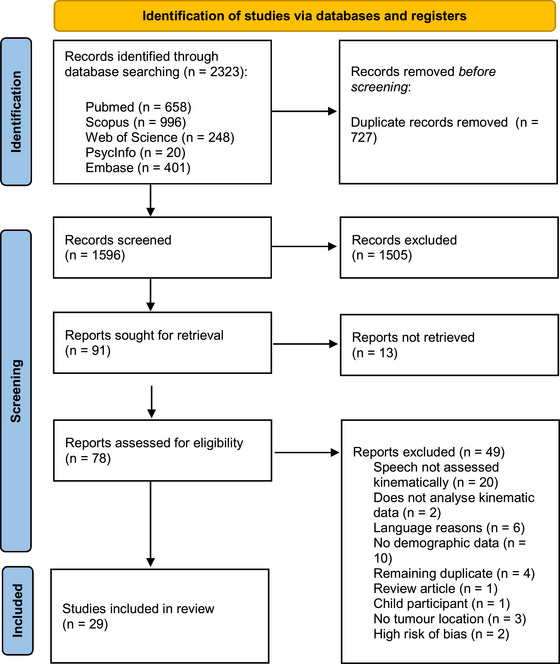
Flow diagram for study selection.

The systematic search through the five databases yielded 2323 records. A total of 727 records were identified as duplicates and a further 1505 records were removed during abstract screening. As 13 reports were not retrievable, the full‐text of 78 items were screened, out of which a further 49 were excluded (for exclusion reasons, see Figure 1). Two studies were excluded because of high risk of bias as they scored less than 50% on the critical appraisal lists (Bressmann et al., 2014; Quintero et al., 2009). This resulted in 29 studies included in the review.

### Study characteristics

#### Patient and tumour characteristics

The 29 studies included a total of 197 individuals treated for O&OSCC and 185 control speakers. A total of 12 studies included only individuals with O&OSCC (Acher et al., 2014; Davis et al., 1987; Georgian et al., 1982; Hagedorn et al., 2014, 2022; Imai & Michi, 1992; Mady & Beer, 2007; Morrish, 1988, 1984; Schliephake et al., 1998; Suzuki, 1989; Wakumoto et al., 1996) and 17 studies included typical speakers as well (Barry & Timmermann, 1985; Bressmann, Thind et al., 2005; Bressmann, Uy et al., 2005; Bressmann et al., 2007; Fletcher, 1988; Grimm et al., 2017; Ha et al., 2016; Hagedorn et al., 2021; Hamlet et al., 1990, 1992; Kansy et al., 2017, 2018; Rastadmehr et al., 2008; Stone et al., 2014, 2012; Yoshioka et al., 2004; Zhou et al., 2013). The median number of individuals with O&OSCC was five, with only six studies including more than 10 individuals with O&OSCC (Bressmann et al., 2007; Grimm et al., 2017; Ha et al., 2016; Imai & Michi, 1992; Schliephake et al., 1998; Stone et al., 2012). The majority of studies included small and heterogeneous patient groups, and unmatched control groups in terms of age. For example, the mean age of the included individuals treated for O&OSCC was 53 and 35 years for the typical speakers. The mean difference between speaker groups was 16.6 years (SD = 15.2 years). One study did not report the age of both speaker groups, but did report speaker sex (Zhou et al., 2013). One study did not report the age of the control speakers (Barry & Timmermann, 1985). Two studies did not report the sex of both speaker groups, but did report speaker age (Rastadmehr et al., 2008; Stone et al., 2014). One study did not mention the sex of the control speakers (Barry & Timmermann, 1985). Eleven studies did not explicitly mention the TNM staging of the individuals with O&OSCC (Barry & Timmermann, 1985; Bressmann, Thind et al., 2005; Bressmann, Uy et al., 2005; Bressmann et al., 2007; Davis et al., 1987; Georgian et al., 1982; Hagedorn et al., 2014; Imai & Michi, 1992; Morrish, 1988, 1984; Suzuki, 1989). Out of these 11 studies, three studies specified the extent of the resection by means of a schematic (Bressmann et al., 2007; Imai & Michi, 1992; Suzuki, 1989); two provided the percentage‐of‐tongue‐removed (Barry & Timmermann, 1985; Georgian et al., 1982); four studies specified that the individual underwent (sub)total glossectomy (Bressmann, Uy et al., 2005; Davis et al., 1987; Morrish, 1988, 1984); one study used the adjectives ‘advanced’, ‘small’ or ‘medium’ (Hagedorn et al., 2014); and one study did not specify the size of the resection (Bressmann, Thind et al., 2005). All other studies specifically mentioned the TNM staging. Four studies included individuals who received speech therapy (Davis et al., 1987; Georgian et al., 1982; Morrish, 1988, 1984); nine studies explicitly stated that the individuals had not received speech therapy (Barry & Timmermann, 1985; Hagedorn et al., 2021, 2014, 2022; Hamlet et al., 1990, 1992; Imai & Michi, 1992; Stone et al., 2014; Suzuki, 1989); and the other 16 studies did not specify information regarding speech therapy.

Across the 29 studies and 197 speakers included in the review, most individuals were treated for a tumour located on the anterior two‐thirds and/or posterior one‐third of the tongue (*n* = 134, 62.9%) or on the floor of the mouth (*n* = 47, 22.1%). Five individuals (2.4%) were treated for a tumour on the tongue in combination with the mandible, and 14 in combination with the floor of the mouth (6.6%), or both the mandible and floor of the mouth (*n* = 5, 2.4%). Finally, three individuals (1.4%) were treated for a tumour on the floor of the mouth with mandibular involvement. No individuals were treated for solely a mandibular tumour. An overview of the summary statistics of the included summaries is provided in Table 2.

**TABLE 2:** Overview of study characteristics.

Review criteria	Results	
**Type of study**		**Count**
Prospective		13
Retrospective		16
**Patient characteristics**		**Range (mean, SD)**
Sample size	Prospective	1–40 (M = 7.8; SD = 7.4)
	Retrospective	1–17 (M = 5.9, SD = 8.7)
Time post‐treatment	Prospective	1–12 months
	Retrospective	1–120 months
**Tumour and treatment (count)**		**Count (prospective, retrospective)**
TNM staging (*n* = 21)	T1	45 (P = 17, R = 28)
	T2	53 (P = 30, R = 23)
	T3	22 (P = 16, R = 6)
	T4	24 (P = 21, R = 3)
Localization (*n* = 29)	Anterior two‐thirds of the tongue	78 (P = 17, R = 61)
	Posterior one‐third of the tongue	15 (P = 2, R = 13)
	Anterior two‐thirds + posterior one‐third	25 (P = 11, R = 14)
	Mandible	0 (P = 0, R = 0)
	Floor of mouth	47 (P = 47, R = 0)
	Tongue + mandible	10 (P = 5, R = 5)
	Tongue + floor of mouth	14 (P = 12, R = 2)
	Tongue + mandible + floor of mouth	5 (P = 2, R = 3)
	Mandible + floor of mouth	3 (P = 3, R = 0)
Treatment modality	Surgery	29
	Primary radiation	0
PORT	Yes	7 (P = 3, R = 4)
	No	22 (P = 10, R = 12)
**Methodology**		**Count (prospective, retrospective)**
Recording type	UTI	7 (P = 7, R = 0)
	MRI	10 (P = 2, R = 8)
	Videofluoroscopy	6 (P = 2, R = 4)
	EPG	5 (P = 2, R = 3)
	Pressure sensor	1 (P = 0, R = 1)
Type of task	Phoneme repetition	4 (P = 4, R = 0)
	Syllable repetition	5 (P = 2, R = 3)
	Word repetition	11 (P = 6, R = 5)
	Phrase repetition	5 (P = 0, R = 5)
	Passage reading	4 (P = 1, R = 3)

*Note*: EPG, electropalatography; M, mean; MRI, magnetic resonance imaging; P, prospective; PORT, postoperative radiation therapy; R, retrospective; SD, standard deviation; UTI, ultrasound tongue imaging.

#### Study characteristics and imaging techniques

Of the 29 studies, 13 were prospective (Acher et al., 2014; Bressmann, Thind et al., 2005; Bressmann, Uy et al., 2005; Bressmann et al., 2007; Fletcher, 1988; Hamlet et al., 1990, 1992; Kansy et al., 2017, 2018; Mady & Beer, 2007; Rastadmehr et al., 2008; Schliephake et al., 1998; Wakumoto et al., 1996) and 16 retrospective (Barry & Timmermann, 1985; Davis et al., 1987; Georgian et al., 1982; Grimm et al., 2017; Ha et al., 2016; Hagedorn et al., 2021, 2014, 2022; Imai & Michi, 1992; Morrish, 1988, 1984; Stone et al., 2014, 2012; Suzuki, 1989; Yoshioka et al., 2004; Zhou et al., 2013). For the prospective studies, four studies were cohort studies (Acher et al., 2014; Mady & Beer, 2007; Schliephake et al., 1998; Wakumoto et al., 1996) and nine studies included a cohort together with a control group (Bressmann, Thind et al., 2005; Bressmann, Uy et al., 2005; Bressmann et al., 2007; Fletcher, 1988; Hamlet et al., 1990, 1992; Kansy et al., 2017, 2018; Rastadmehr et al., 2008). For the retrospective studies, seven employed a case‐control design (Grimm et al., 2017; Ha et al., 2016; Hagedorn et al., 2021; Stone et al., 2014, 2012; Yoshioka et al., 2004; Zhou et al., 2013), six reported a case series (Barry & Timmermann, 1985; Hagedorn et al., 2014, 2022; Imai & Michi, 1992; Morrish, 1984; Suzuki, 1989), and three reported on a single individual (Davis et al., 1987; Georgian et al., 1982; Morrish, 1988). Three studies appeared in conference proceedings (Hagedorn et al., 2014; Mady & Beer, 2007; Zhou et al., 2013) and 26 were published as journal articles. All articles were published between 1982 and 2022.

A total of 10 studies quantified speech parameters by using a form of magnetic resonance imaging (MRI; e.g., cine‐MRI or rt‐MRI; Grimm et al., 2017; Ha et al., 2016; Hagedorn et al., 2021, 2014, 2022; Kansy et al., 2017; Mady & Beer, 2007; Stone et al., 2014, 2012; Zhou et al., 2013). Seven studies employed 2D or 3D ultrasound tongue imaging (UTI; Acher et al., 2014; Bressmann, Thind et al., 2005; Bressmann, Uy et al., 2005; Bressmann et al., 2007; Kansy et al., 2018; Rastadmehr et al., 2008; Schliephake et al., 1998). Six studies used videofluoroscopy (Davis et al., 1987; Georgian et al., 1982; Hamlet et al., 1990, 1992; Morrish, 1988, 1984). Five studies used electropalatography (EPG; Barry & Timmermann, 1985; Fletcher, 1988; Imai & Michi, 1992; Suzuki, 1989; Wakumoto et al., 1996), and one study used a pressure sensor to quantify speech (Yoshioka et al., 2004). Studies were conducted by research groups in six countries. A total of 13 studies were conducted in the United States (Davis et al., 1987; Fletcher, 1988; Georgian et al., 1982; Grimm et al., 2017; Ha et al., 2016; Hagedorn et al., 2021, 2014, 2022; Hamlet et al., 1990, 1992; Stone et al., 2014, 2012; Zhou et al., 2013), four in Canada (Bressmann, Thind et al., 2005; Bressmann, Uy et al., 2005; Bressmann et al., 2007; Rastadmehr et al., 2008), five in Germany (Barry & Timmermann, 1985; Kansy et al., 2017, 2018; Mady & Beer, 2007; Schliephake et al., 1998), four in Japan (Imai & Michi, 1992; Suzuki, 1989; Wakumoto et al., 1996; Yoshioka et al., 2004), two in the UK (Morrish, 1988, 1984), and one in France (Acher et al., 2014).

#### Quality of evidence and risk of bias

The quality rating of the body of evidence (GRADE) for the final number of included studies was ‘low’ or ‘very low’ for all studies. Risk of bias was judged to be low (80% or higher) for four studies, moderate (66–80%) for 14 studies, and high (50–65%) for 11 studies. An overview of the characteristics of individual studies, paired with their main finding, is provided in Table 3.

**TABLE 3:** Overview of individual studies, including the study type, risk of bias, number of individuals treated for O&OSCC, T‐stages of the individuals, used method, task (phoneme, syllable, word, or passage repetition), time of evaluation, and the main finding.

Study	Study type	RoB	*N*	T‐stage	Method	Task	Evaluation time(s)	Main finding
Acher et al. (2014)	P	H	2	T4	UTI	Syllable	Pre‐ and 1–3 months	Individuals treated for O&OSCC had decreased capacity to shape the entire tongue correctly which suggests a global stiffening.
Barry and Timmermann (1985)	R	H	7	–	EPG	Passage	–	Difficulty in making alveolar or velar contact. Plosives may turn into fricatives due to air leakage.
Bressmann, Thind et al. (2005)	P	H	1	–	UTI	Phoneme	Pre‐ and 4 weeks	More asymmetrical movement post‐surgery compared with pre‐surgery. More concave tongue shape post‐surgery compared with pre‐surgery.
Bressmann, Uy et al. (2005)	P	H	3	–	UTI	Phoneme	Pre‐ and 1 month	Reconstructed segments are not functionally integrated with the rest of the tongue and move passively.
Bressmann et al. (2007)	P	M	12	–	UTI	Phoneme	Pre‐ and 6–9 weeks	Reconstructed (RFFF) individuals treated for O&OSCC had more concave tongue shapes compared with directly closed individuals. All treated individuals had more asymmetric movement compared with controls.
Davis et al. (1987)	R	M	1	–	VF	Word	5–12 months	Palatal prosthesis facilitated palatal contact for alveolar plosives for low vowels. Bilabial compensation for alveolar and velar plosives without prosthesis.
Fletcher (1988)	P	H	3	T2–T3	EPG	Word	Pre‐ and 1–3 months (P) Between 4 and 12 months (R)	All individuals treated for O&OSCC showed increased contact area in between sessions for /s/. One individual showed labial gestures for alveolar /t, d/.
Georgian et al. (1982)	R	L	1	–	VF	Word	–	Inability to maintain complete closure. Lingual‐velar contact for alveolar /t,d/ with occasional bilabial contact.
Grimm et al. (2017)	R	L	14	T1–T2	MRI	Phrase	Between 9 months and 8 years	Individuals treated for O&OSCC are more likely to use laminal /s/ instead of apical /s/ compared with controls.
Ha et al. (2016)	R	M	13	T1–T2	MRI	Phrase	Between 6 months and 4 years	Individuals treated for O&OSCC have more asymmetric tongue movement during /i/ and /u/ and a slightly flatter tongue position compared with controls.
Hagedorn et al. (2014)	R	H	5	–	MRI	Word	At least 4 months	Labiodental and dorsal constrictions to produce alveolar stops and laterals. Dorsal frication for alveolar /s/.
Hagedorn et al. (2021)	R	H	6	T2–T4	MRI	Passage	At least 4 months	Individuals treated for O&OSCC showed less complex vocal tract shaping. Two individuals showed reduced changes in velar and alveolar regions.
Hagedorn et al. (2022)	R	H	2	T3–T4	MRI	Passage	At least 6 months	Compensatory strategies to produce target alveolar segments vary systematically as a function of target manner. Alveolar targets are replaced by either labial or velar constrictions of varying magnitude.
Hamlet et al. (1990)	P	M	5	T2–T3	VF	Word	Pre‐ and 4–5 months	All individuals treated for O&OSCC had difficulty in moving the tongue backwards. More mobility (i.e., larger differentiation) between consonant vowel pairs post‐radiation compared with post‐surgery.
Hamlet et al. (1992)	P	M	5	T2–T3	VF	Word	Pre‐, 2–7 weeks following surgery and 4–10 weeks following radiation	Higher jaw posture to increase *F*_2_ for /u/, but contribution was minor.
Imai and Michi (1992)	R	M	17	–	EPG	Syllable	Between 6 and 12 months	/t/ was perceptually the most distorted and was produced with partial constriction. areas with more than a few contacts produced less distorted sounds for /s/.
Kansy et al. (2017)	P	M	1	T2	MRI	Word	Pre‐ and 1, 3, 6, 12 months	Increased distance between most anterior caudal point of the tongue and tongue tip, dorsum and back for most sounds 1 month post‐surgery compared with pre‐surgery.
Kansy et al. (2018)	P	M	1	T2	UTI	Word	Pre‐ and 1, 3, 6, 12 months	Increased distance between most anterior caudal point of the tongue and tongue tip, dorsum and back for most sounds 1 month post‐surgery compared with pre‐surgery.
Mady and Beer (2007)	P	L	8	T1–T2	MRI	Word	1 month	Lateralization of /S/ and palatalization of /s/. More severely affected individuals treated for O&OSCC could not produce /x/.
Morrish (1984)	R	H	2	–	VF	Word	72–120 months	Jaw protrusion for front vowels and retracted more for back vowels. Velar closure used to preserve acoustic loss through velo‐pharyngeal port.
Morrish (1988)	R	M	1	–	VF	Word	8 years	All plosives produced with bilabial seal, but significantly lower jaw for bilabials compared with alveolars. Compensatory movement had no effect on acoustics or perception. Compensatory jaw lowering for vowels.
Rastadmehr et al. (2008)	P	H	10	T1–T3	UTI	Passage	Pre‐ and 2 months	Individuals treated for O&OSCC had higher tongue velocity post‐surgery compared with controls.
Schliephake et al. (1998)	P	L	40	T1–T4	UTI	Phoneme	Pre‐ and 6 months	Significantly reduced tongue mobility and speech quality. Weak but significant correlation between mobility and intelligibility.
Stone et al. (2012)	R	M	15	T1–T2	MRI	Phrase	At least 8 months	Apical /s/ is less frequently used by individuals treated for O&OSCC compared with controls. Shallower groove during /s/ compared with controls.
Stone et al. (2014)	R	M	3	T1	MRI	Phrase	Between 6 and 47 months	More uniform tongue tip and body movement in individuals treated for O&OSCC along the midline. No evidence for compensatory behaviour on the non‐tumour side.
Suzuki (1989)	R	M	3	–	EPG	Syllable	2 months	2 months post‐surgery, the linguopalatal contact was restricted. Asymmetrical contact patterns during /s/
Wakumoto et al. (1996)	P	M	10	T1–T4	EPG	Syllable	Pre‐ and 1, 6, 12 months	Reconstructed individuals had more dental arch contact than directly sutured individuals. More mid‐palatal contact resulted in reduced intelligibility.
Yoshioka et al. (2004)	R	M	5	T1–T4	Pressure sensor	Syllable	Between 6 and 120 months	Reduced alveolar consonantal contrast in terms of pressure against the palate in individuals treated for O&OSCC.
Zhou et al. (2013)	R	H	2	T2	MRI	Phrase	–	More posterior constriction for /s/ for individuals treated for O&OSCC. Similar grooving for /s/ and /S/ for individuals treated for O&OSCC, but not controls.

*Note*: EPG, electropalatography; H, High; L, Low; M, Moderate; MRI, magnetic resonance imaging; N, number of individuals treated for, O&OSCC; O&OSCC oral or oropharyngeal squamous cell carcinoma; P, prospective; R, retrospective; RoB, Risk of Bias; UTI, ultrasound tongue imaging; VF, videofluoroscopy.

### Results of synthesis

#### Articulatory–kinematics affected by treatment

##### Global tongue movement

Five studies assessed global tongue movement in individuals with O&OSCC using different methods (Acher et al., 2014; Hagedorn et al., 2021; Rastadmehr et al., 2008; Schliephake et al., 1998; Stone et al., 2014). One study (Acher et al., 2014) calculated the ‘Speed Normalized Tongue Surface’ which assesses the speed and direction of tongue movements based on UTI data of CVCVC sequences (C = consonant, V = vowel). At 3 months following surgery and radiation, individuals treated for tongue cancer showed a flattening of the tongue surface compared with pre‐surgery, indicating a stiffening of the tongue and general difficulty with shaping the tongue correctly. One UTI study (Schliephake et al., 1998) reported reduced overall tongue mobility in millimetres following surgery for floor‐of‐mouth tumours compared with pre‐surgery during phoneme repetition. The last UTI study (Rastadmehr et al., 2008) showed a higher tongue velocity during a reading passage following surgery for tongue cancer compared with pre‐surgery while speaking rate remained similar, signalling compensatory behaviour. However, an MRI study using phrase repetition did not find any compensatory behaviour when looking at the movement profiles of the affected and non‐affected side of the tongue in individuals post‐surgery. This study further showed that individuals treated for tongue cancer showed more uniform movement between the tongue tip and blade compared with control speakers. Thus, the tongue tip moved less independently in individuals treated for tongue cancer compared with control speakers (Stone et al., 2014). Utilizing a principal component analysis (PCA) on MRI data of the entire vocal tract during a reading passage, one study (Hagedorn et al., 2021) found that individuals treated for tongue cancer required fewer PCA components to explain the data compared with control speakers, suggesting that treated individuals had less complex vocal tract shaping. This was further shown by the fact that more global forward and backward motion explained more variation in the data of individuals treated for tongue cancer compared with controls than more subtle tongue movements. Overall, the studies suggest that differential control of subparts of the tongue is compromised following surgical treatment for tongue cancer, with conflicting evidence surrounding the compensatory use of the unaffected side.

##### Symmetry

Three studies reported on the symmetry, measured as the difference between the movement of the affected and unaffected side of the tongue, using either phrase repetition with MRI (Ha et al., 2016) or sustained phonemes with UTI (Bressmann, Thind et al., 2005; Bressmann et al., 2007). All three studies found that individuals treated for tumours located on the tongue with and without mandibular involvement showed more asymmetrical tongue movements post‐surgery compared with pre‐surgery (Bressmann, Thind et al., 2005) or post‐surgery compared with control speakers (Bressmann et al., 2007; Ha et al., 2016).

##### Concavity

Five studies assessed concavity of the tongue (i.e., the ability to produce a midsagittal groove) in individuals being treated for O&OSCC (Bressmann, Thind et al., 2005; Bressmann et al., 2007; Ha et al., 2016; Stone et al., 2012; Zhou et al., 2013). Two studies used a concavity index based on UTI data of sustained phonemes in individuals treated for tumours located on the tongue with and without mandibular involvement, which assessed how convex or concave the tongue is along the tongue's entire length during phoneme repetition (Bressmann, Thind et al., 2005; Bressmann et al., 2007). The first study reported a more concave post‐surgery compared with pre‐surgery (Bressmann, Thind et al., 2005). The second study reported a more concave tongue following surgery compared with control speakers for flap reconstructed individuals only (Bressmann et al., 2007). Two MRI studies assessed tongue grooving during /s/ production and found reduced tongue grooving in individuals surgically treated for tongue cancer compared with control speakers (Stone et al., 2012; Zhou et al., 2013). Finally, one MRI study (Ha et al., 2016) assessed tongue grooving during /i/ and /u/ and reported a slightly flatter tongue position for individuals treated for tumours on the tongue compared with control speakers. Overall, problems with proper grooving may be present in individuals treated for tumours located on the tongue for specific phonemes (e.g., /s/ or the vowels /i/ and /u/).

##### Alveolar gestures

Articulatory–kinematics in the alveolar region were investigated in 18 studies using various methods (Barry & Timmermann, 1985; Davis et al., 1987; Fletcher, 1988; Georgian et al., 1982; Grimm et al., 2017; Hagedorn et al., 2021, 2014, 2022; Imai & Michi, 1992; Kansy et al., 2017, 2018; Mady & Beer, 2007; Morrish, 1988; Stone et al., 2012; Suzuki, 1989; Wakumoto et al., 1996; Yoshioka et al., 2004; Zhou et al., 2013). Using a PCA based on whole vocal tract MRI data during a reading passage, one study (Hagedorn et al., 2021) showed reduced movement amplitude in anterior regions in individuals treated for tumours on the oral tongue compared with control speakers. Two studies measured the distance between specific points of the tongue in rest and during the production of alveolar segments using MRI and UTI in both control speakers and an individual treated for a floor‐of‐mouth tumour. These studies found an increased distance between the most anterior caudal point of the tongue and the tongue tip, dorsum and back for all alveolar sounds 1 month post‐surgery compared with pre‐surgery and controls; however, the distances between the anterior caudal point of the tongue and tongue tip returned close to preoperative values at 12 months post‐surgery (Kansy et al., 2017, 2018).

In the context of /s/ production, a retrospective case series EPG study (Suzuki, 1989) showed asymmetric or reduced anterior contact patterns while producing syllables containing /s/ following surgery for tumours located on the tongue with mandibular involvement. Two MRI studies (Grimm et al., 2017; Stone et al., 2012) showed that individuals surgically treated for tongue cancer produce laminal /s/, to a greater extent than control speakers, as laminal /s/ requires less tongue tip raising compared with apical /s/. Three other MRI studies reported on individuals with tongue and/or floor‐of‐mouth tumours and reported a posterior shift in the constriction location of /s/ (i.e., a post‐alveolar or palatal constriction) post‐surgery compared with controls (Zhou et al., 2013), compared with pre‐surgery (Mady & Beer, 2007), or based on a single post‐surgery and radiation recording session (Hagedorn et al., 2014).

For alveolar plosives (/t, d/), four EPG studies reported on data from a single post‐surgery recording session and showed that individuals treated for either tongue tumours with and without mandibular involvement have a partial constriction due to incomplete palatal contact (Barry & Timmermann, 1985; Imai & Michi, 1992; Suzuki, 1989; Wakumoto et al., 1996). Three case reports and two case series providing data from post‐treatment recording sessions reported that individuals who underwent a partial or subtotal glossectomy produced alveolar plosives as bilabials (i.e., with lip closure), both during sentence reading and syllable repetition (Davis et al., 1987; Fletcher, 1988; Georgian et al., 1982; Hagedorn et al., 2022; Morrish, 1988). Additionally, in two of the aforementioned studies, the data showed that alveolar plosives produced by individuals who underwent a partial or subtotal glossectomy might be produced using a velar constriction instead (Georgian et al., 1982; Hagedorn et al., 2022). Finally, a pressure sensor study (Yoshioka et al., 2004) reported no significant pressure and durational differences between /t, ʃ, tʃ/ for individuals surgically treated for tumours located on the tongue while significant differences were found for control speakers (/t/ > /tʃ/ > /ʃ/). However, the interaction between phoneme and group was not formally tested, making it impossible to verify whether the differences between groups were significant. Overall, the findings collectively suggest issues with anterior tongue raising for individuals with O&OSCC post‐surgery, potentially leading to compensatory bilabial or velar productions. The consistency of these observations across diverse measurement methods and comparators (i.e., both compared with pre‐surgery and to control speakers) underscores the robustness of the identified patterns.

##### Velar gestures

Six studies investigated the articulatory–kinematics in the velar region using various methods (Barry & Timmermann, 1985; Hagedorn et al., 2021; Hamlet et al., 1990; Kansy et al., 2017, 2018; Mady & Beer, 2007). One videofluoroscopy study (Hamlet et al., 1990) reported that individuals treated for tumours on the tongue with and without mandibular involvement through surgery and postoperative radiation therapy (PORT) had an overall difficulty with moving the tongue in a posterior direction compared with pre‐surgery in a word repetition task. One MRI study (Hagedorn et al., 2021) used a PCA based on whole vocal tract MRI data during a reading passage and reported reduced movement in the velar region for individuals treated for base of tongue cancer compared with control speakers. In an MRI study of post‐surgery outcomes in speakers with tumours located on the floor‐of‐mouth or tongue, seven of eight speakers were still able to normally produce /x/ following surgery, while one individual showed a moderate impairment of /x/, showing a longer constriction length following surgery (Mady & Beer, 2007). One EPG study (Barry & Timmermann, 1985) reported difficulty of making velar contact during a reading passage in individuals surgically treated for tumours located on the tongue compared with reference values. Finally, two studies measured the distance between specific points of the tongue in rest and during the production of velar segments using MRI and UTI in both control speakers and an individual treated for a floor‐of‐mouth tumour. Both studies reported an increased distance between the most anterior caudal point of the tongue and the tongue tip, dorsum and back for velar sounds 1 month post‐surgery compared with pre‐surgery and controls, but the distances return close to preoperative values at 12 months post‐surgery for plosives /k, g, ŋ /, though /k, ŋ/ do not fall within typical ranges (Kansy et al., 2017, 2018). Overall, results show a difficulty with raising the back part of the tongue appropriately in order to hit velar targets in individuals treated for O&OSCC, either compared with pre‐surgery recordings or to control speakers.

##### Vowels

Eight studies investigated the articulatory–kinematics of vowels using a variety of methods (Bressmann, Thind et al., 2005; Ha et al., 2016; Hamlet et al., 1992; Kansy et al., 2017, 2018; Morrish, 1988, 1984). Three studies assessed compensatory movement in vowel production following partial or (sub)total glossectomy using both videofluoroscopy and speech acoustics (Hamlet et al., 1992; Morrish, 1988, 1984). While vowel height (as measured by the acoustic first formant: *F*_1_) was well preserved, the front–back distinction (as measured by the second formant: *F*_2_) was heavily reduced. To discriminate between front and back vowels, the jaw was protruded for front vowels and retracted for back vowels (Morrish, 1984). Compensatory jaw movement could result in both raising (Hamlet et al., 1992) and lowering (Morrish, 1988, 1984). The velum was raised to prevent acoustic loss through the nasal cavity (Morrish, 1984). Lastly, lip rounding was employed to reduce the *F*_2_ of /u/ to help distinguish it from /i/ (Morrish, 1988). One MRI study (Ha et al., 2016) assessed the symmetry of tongue movements during /i/ and /u/ following surgery for a tumour located on the tongue and found an increase in asymmetrical movement for treated individuals compared with control speakers. One study (Bressmann, Thind et al., 2005) provided qualitative descriptions of 3D ultrasound data of the corner vowels /i, a, u/ and reported a flatter tongue following surgery compared with pre‐surgery in a single individual following surgery for a tumour on the tongue with mandibular involvement. Lastly, two studies assessed the distance between various points of the tongue during rest and the production of vowels using MRI and UTI in both control speakers and an individual treated for a floor‐of‐mouth tumour. These studies found an increased distance 1 month post‐surgery compared with pre‐surgery and control speakers, but these distances moved towards pre‐surgery levels at the 12‐month follow‐up (Kansy et al., 2017, 2018). Overall, movement is most restricted in the anteroposterior direction following treatment for O&OSCC, for which individuals created varying compensatory strategies.

#### Effect of clinical variables

##### TNM staging

To assess kinematic changes in relation to TNM staging, we only synthesize studies that (1) provided the TNM staging of the participant and (2) included participants with varying TNM staging. This resulted in a subset of 13 studies (44.8%). Of these 13 studies, eight commented on either individual data or patterns based on TNM staging (Fletcher, 1988; Ha et al., 2016; Hagedorn et al., 2021; Hamlet et al., 1990, 1992; Mady & Beer, 2007; Stone et al., 2012; Wakumoto et al., 1996). Five did not analyse TNM staging directly (Grimm et al., 2017; Hagedorn et al., 2022; Rastadmehr et al., 2008; Schliephake et al., 1998; Yoshioka et al., 2004).

Six studies found that individuals with smaller tumours had better kinematic speech outcomes post‐surgery compared with individuals with larger tumours (Fletcher, 1988; Ha et al., 2016; Hagedorn et al., 2021; Hamlet et al., 1992; Mady & Beer, 2007; Stone et al., 2012). Individuals treated for T2 tumours had more typical contact patterns for /t/ and grooving for /s/ compared with those who were treated for T3 tumours based on EPG and videofluoroscopic data (Fletcher, 1988; Hamlet et al., 1992). A whole tract MRI study (Hagedorn et al., 2021) reported that the individual who was treated for a T2 tumour had more complex vocal tract shaping compared with those treated for larger tumours (T3–T4) as those with larger tumours needed fewer principal components to explain 99% variance in the data than the individual who was treated for a T2 tumour. An MRI study (Ha et al., 2016) reported that individuals treated for T1 tumours had more typical back cavity lengths during vowel production compared with individuals treated for T2 tumours, signalling more typical tongue placement. Another MRI study (Mady & Beer, 2007) showed that the vocal tract shaping of individuals treated for T1 tumours was affected less during the production of /s, ʃ, l/ compared with individuals with T2 tumours following treatment. Lastly, individuals treated for T1 tumours had more pronounced tongue grooving during /s/ compared with individuals treated for T2 tumours based on MRI data (Stone et al., 2012).

Two studies did not find evidence for different patterns between the articulatory patterns of individuals with varying TNM staging. A videofluoroscopy study (Hamlet et al., 1990) reported no evidence for different articulatory patterns of individuals treated for T2 and T3 tumours during nonce word repetition. An EPG study (Wakumoto et al., 1996) reported widely varying contact patterns during /ta/ in individuals treated for T1 through T4 tumours that were not directly related to tumour size, but more to other clinical variables such as reconstruction method. For example, an individual with a smaller resection that was locally closed had a partial constriction for /t/, whereas an individual with a larger resection and flap reconstruction achieved full constriction. Overall, despite some variability, smaller tumours lead to more typical or less affected movement patterns compared with larger tumours in individuals treated for O&OSCC.

##### Tumour location

To assess kinematic changes in relation to the location of the tumour, we only synthesize studies that included participants with varying tumour locations which resulted in a subset of 15 studies (51.7%). Of these 15 studies, 10 commented on individual data or patterns based on tumour location (Barry & Timmermann, 1985; Bressmann, Uy et al., 2005; Bressmann et al., 2007; Fletcher, 1988; Hagedorn et al., 2021, 2014, 2022; Imai & Michi, 1992; Mady & Beer, 2007; Schliephake et al., 1998). Four studies did not comment on individual data or location data specifically (Hamlet et al., 1990, 1992; Rastadmehr et al., 2008; Yoshioka et al., 2004). One study (Morrish, 1984) only analysed the kinematic data of one of the two speakers, making it impossible to compare between tumour locations.

Five studies found reduced or altered movement at the site of resection compared with surrounding structures following surgery (Barry & Timmermann, 1985; Bressmann, Uy et al., 2005; Hagedorn et al., 2021, 2014, 2022). Specifically, one MRI study (Hagedorn et al., 2014) found that an individual with a tumour on the oral tongue produced /s/ with the tongue dorsum whereas the individual treated for base of tongue cancer produced /s/ using the tongue tip. Using a PCA based on whole vocal tract MRI data during a reading passage, one study (Hagedorn et al., 2021) found reduced amplitude in the affected region compared with the unaffected regions. That is, they reported reduced movement in the alveolar region for tumours on the oral tongue and reduced velar movement for base of tongue tumours. One MRI study (Hagedorn et al., 2022) assessed compensatory strategies following oral or oral and base of tongue resections and found that individuals used the unaffected part of the tongue in a compensatory manner for sounds that are typically produced with the affected part of the tongue. For example, an individual treated for a tumour on the oral tongue produced alveolar plosives with a velar or bilabial constriction. One retrospective case series (Barry & Timmermann, 1985) assessed palatal contact patterns with EPG and found that while individuals treated for anterior tumours could not make alveolar contact during /t/, palatal contact for /k/ was possible. Finally, one study (Bressmann, Uy et al., 2005) conducted a whole tongue PCA analysis of UTI data and found that affected areas moved differently from the unaffected areas. That is, the affected area of the tongue comprised its own principal component.

While two other studies did find that movement and contact patterns varied according to tumour site (i.e., reduced movement in affected areas compared with unaffected areas), tumour site was correlated with other clinical variables like tumour size or reconstruction method, making it impossible to determine the cause of the established pattern (Bressmann et al., 2007; Fletcher, 1988). In general, there is a great confound between tumour size, reconstruction method, and the possibility of an individual receiving PORT. For example, individuals with larger tumours are more likely to be reconstructed and receive PORT than individuals with smaller tumours. Thus, in order to assess the effect of the reconstruction method in isolation, a carefully matched participant group would be required.

One study (Schliephake et al., 1998) assessed tongue mobility before and after surgery for floor of mouth carcinomas using UTI and reported that median and bilateral tumours resulted in the most severely reduced mobility whereas lateral tumours had the smallest effect compared with pre‐surgery mobility.

Lastly, two studies did not find direct evidence for the effect of tumour location on kinematic speech outcomes. One MRI study (Mady & Beer, 2007) assessed the vocal tract shaping during the production of /s, ʃ, l, x/ and found that tumour location (anterior tongue or floor of mouth) was not predictive for /s, ʃ, l, x/. However, an intact genioglossus muscle and no fixation of the tongue to the floor‐of‐mouth predicted typical /l/ production. One retrospective case series (Imai & Michi, 1992) reported highly variable EPG contact patterns and duration for /t, s, ʃ, ç/ within tumour groups (anterior and anterior–posterior tongue). Despite individual variation, the overall evidence suggests that tumour location may predict kinematic changes such that phonemes whose place of articulation corresponds to the tumour location might be affected in individuals treated for O&OSCC.

##### Primary treatment modality

This question could not be answered as all studies included only individuals with O&OSCC who received surgical treatment as opposed to primary (chemo)radiation.

#### Effect of adjuvant radiation therapy

This question could not be answered as studies who included individuals with O&OSCC who received adjuvant radiation therapy (*n* = 7, 24.1%) either included only individuals who received adjuvant radiation therapy radiation (Acher et al., 2014; Hagedorn et al., 2021, 2014, 2022; Hamlet et al., 1990, 1992), or did not comment on radiation based differences (Grimm et al., 2017).

#### Speech outcomes over time post‐treatment

To assess articulatory–kinematic speech outcomes over time, post‐surgical treatment, only studies that tested participants multiple times following surgery were included in this part of the synthesis. Of the longitudinal studies (*n* = 13), seven studies included multiple measurements post‐surgery (Acher et al., 2014; Fletcher, 1988; Hamlet et al., 1990, 1992; Kansy et al., 2017, 2018; Wakumoto et al., 1996). However, one study (Wakumoto et al., 1996) collected kinematic data at one time point post‐surgery while acoustic and perceptual data was collected at multiple time points, and was therefore excluded from this part of the synthesis.

Two studies assessed the change between various points of the tongue during rest and the production of the sounds of German in an individual treated for a floor‐of‐mouth tumour at 1, 3, 6 and 12 months following surgery using the same protocol with both MRI and UTI (Kansy et al., 2017, 2018). The results showed an increase in distance for all phonemes attributable to postoperative swelling and tissue edema. While these distances changed in the direction of the pre‐surgery values, some residual elevation was present at 12 months following surgery between the most anterior caudal point and the most dorsal point of the tongue for /x, k, g, r/. Residual elevation between the most anterior caudal point and the most cranial point of the tongue at 12 months following surgery was found for /k, d, e:, f, i, j, l, m, n, ŋ, o:, ø:, r, u:, y:/. Residual elevation at 12 months following surgery between the anterior caudal point and the tongue tip was found for /x, e:, ɛ:. k, d, j, m, n, ø:, p, s, u:, y:/. Two videofluoroscopy studies assessed tongue contour changes in consonant–vowel production 2–7 weeks following surgery and 4–10 weeks following radiation in (Hamlet et al., 1990), with an additional 6‐month follow‐up in Hamlet et al. (1992). Results showed that four out of five individuals treated for O&OSCC showed greater tongue differentiation between the consonant and vowel following radiation compared with post‐surgery in /ki/ and three out of five showed greater vowel differentiation following radiation compared with post‐surgery in /tu/. Moreover, four out of five individuals treated for O&OSCC had more tongue root advancement, leading to more fronting and/or tongue height for /i/ following radiation. In terms of jaw movement, individuals treated for O&OSCC had significantly higher jaw position for /u/ following radiation, and significantly lower jaw position at the 6‐month follow‐up. One EPG study (Fletcher, 1988) assessed contact patterns during /s/ and found an increase in the number of contacted sensors between recording sessions that were spaced 2–3 weeks apart for individuals who received treatment 1–12 months ago. One study (Acher et al., 2014) measured the relative tongue shape changes during CVCVC sequences 1 and 3 months following surgery using UTI. Their results showed a global stiffening, or reduction in strength of the tongue during the production of /asa/ and /isi/ at 3 months compared with 1 month following surgery. Overall, results are highly variable, but preliminary evidence suggests that improvement following radiation therapy may be possible.

## DISCUSSION

The primary goal of this systematic review was to evaluate to what extent treatment for O&OSCC affects the articulatory–kinematics of the tongue, jaw, and lips during speech. Our systematic review consisted of 29 studies with a total of 197 individuals treated for O&OSCC that were published between 1982 and 2022. Most studies included a limited number of individuals treated for O&OSCC, with only six (20.7%) having more than 10 participants. Risk of bias was judged to be moderate to high for 25/29 studies (86.2%), mostly resulting from incomplete patient descriptions, unspecified in‐ and exclusion criteria, and control groups that were not matched on age. No studies assessed kinematic speech outcomes following secondary radiation, but surgery was always the primary treatment. Therefore, the discussed results should be interpreted in context of the literature available at the time of our review.

The first aim of our review was to assess to what extent treatment for oral or oropharyngeal cancer affects the articulatory–kinematics of the tongue, jaw, and lips during speech. The results of our synthesis reveal both global and local articulatory–kinematic changes in individuals who underwent surgical treatment for O&SSC. In terms of global changes, multiple studies reported reduced or less complex articulatory–kinematic patterns following treatment. Smaller movement sizes may result in reduced speech mobility considering there is less distance between individual sounds (Bressmann et al., 2004; Matsui et al., 2007). While intelligibility may be preserved in a smaller articulatory space, phonemes need to be produced within this reduced space with maximal distinction (i.e., maximal distance between individual phonemes in the articulatory working space; Lee & Bell, 2018; Weismer, 2013). However, the articulatory precision it requires may become problematic if articulatory control over different parts of the tongue is reduced or when vocal tract shaping is less complex, resulting in potentially less pronounced differences (Acher et al., 2014; Hagedorn et al., 2021; Stone et al., 2014).

Articulatory–kinematic changes following surgical treatment for tongue tumours with and without mandibular involvement were also observed in terms of movement asymmetry of the affected and unaffected side of the tongue (Bressmann, Thind et al., 2005; Bressmann et al., 2007; Ha et al., 2016). Both flap reconstructions and local closures may result in asymmetrical movement. For flap reconstructions, the flap is not functionally integrated and only moves passively (Bressmann, Uy et al., 2005). For local closures, changes to the musculature and volume of the tongue due to the excision and its resulting scar tissue affect movement symmetry. The extent to which the unaffected side of the tongue compensates remains inconclusive as studies showed both the presence and absence of compensatory behaviour of the unaffected side of the tongue (Rastadmehr et al., 2008; Stone et al., 2014). As both studies looked at (increased) tongue velocity as a compensatory mechanism, future studies should investigate other potential compensatory strategies employed by individuals treated for O&OSCC that were not captured in these studies.

Lastly, the results indicate that individuals surgically treated for O&OSCC experience difficulty in forming a proper groove when producing specific phonemes, most notably during /s/ (Bressmann, Thind et al., 2005; Bressmann et al., 2007; Stone et al., 2012; Zhou et al., 2013). A flatter tongue surface (i.e., reduced grooving) may result in a wider constriction that allows more air to escape under lower levels of pressure, changing the acoustic properties of the phoneme. Observed changes in tongue grooving highlight that not all speech changes induced by O&OSCC treatment derive from reduced gross range of motion. Instead, more finer grained aspects of speech motor control are impacted as well. Rehabilitation wise, these finer grained deficits will most likely benefit from specific exercises rather than global non‐speech oral motor exercises that aim to increase range of motion.

Local, more specific, articulatory–kinematic changes were noted as well, most notably the difficulty in tongue‐tip raising and fronting. The inability to raise the tongue‐tip may result in problems with sounds requiring (near) contact with the alveolar ridge, such as the alveolar sounds /t, d, s, z/ as shown by the EPG studies in our review (Barry & Timmermann, 1985; Fletcher, 1988; Imai & Michi, 1992; Suzuki, 1989; Wakumoto et al., 1996). If a constriction near the alveolar ridge was impossible for the individual, bilabial (Davis et al., 1987; Fletcher, 1988; Georgian et al., 1982; Hagedorn et al., 2022; Morrish, 1988) or velar (Georgian et al., 1982; Hagedorn et al., 2022) constrictions were used instead. Issues with tongue‐tip raising and fronting further resulted in less fronted /s/ production and a general preference for laminal /s/ as opposed to apical /s/ considering that the former requires less tongue‐tip raising (Grimm et al., 2017; Hagedorn et al., 2014; Mady & Beer, 2007; Stone et al., 2012; Zhou et al., 2013). A more posterior realization of /s/ may result in the merging of /s/ and /ʃ/, making them acoustically and perceptually similar (Tienkamp et al., 2023; Zhou et al., 2013). This may negatively affect speaker intelligibility. Lastly, problems with tongue fronting and raising may impede the production of the front high vowel /i/ as the tongue cannot reach a high and fronted enough position for its proper production. Taken together, the literature suggests individuals surgically treated for O&OSCC might raise and protrude the jaw as a compensatory response (Hamlet et al., 1992; Morrish, 1984).

A second local effect of surgical treatment for O&SSC on articulatory–kinematics was related to the tongue movements required to produce phonemes in the velar region. Both individuals treated for anterior and posterior tongue tumours experienced difficulty in curling the tongue back enough to produce the velar targets, but these problems were most pronounced in individuals with posterior tumours (Barry & Timmermann, 1985; Hagedorn et al., 2021; Hamlet et al., 1990). Combined with impaired tongue‐fronting, the results suggest overall problems with movement in the anteroposterior direction and super‐inferior movement of the endpoints of the tongue (tip and back). This seems to conflict with the overall finding that vowel height (as measured by the acoustic first formant: *F*_1_) is preserved in individuals surgically treated for O&OSCC considering it is modulated by tongue height (Hagedorn et al., 2014; Morrish, 1984). However, the height of the jaw may contribute considerably to the *F*_1_ together with the tongue body, rather than the tip or the back of the tongue, which might explain why superior–inferior movement does not seem to be impacted in vowels for individuals surgically treated for O&OSCC when measured acoustically. It is also important to note that there is only an imperfect relationship between vowel formants and vowel height and backness (Kuo & Berry, 2023; Lee et al., 2016; Wieling et al., 2016). This further underlines the importance of characterizing the speech of individuals treated for O&OSCC using articulatory–kinematic methods in addition to acoustic or perceptual methods as mobility impairments might be masked in acoustic and perceptual appraisals due to compensatory strategies. Moreover, kinematic data might be especially effective in designing specific exercises that target the locus of the problem. Currently, speech–language pathologists (SLPs) treating individuals who underwent treatment for O&OSCC focus on compensatory articulatory–kinematic strategies rather than active rehabilitation due to a lack of relevant evidence‐based rehabilitation methods (Blyth et al., 2024). If active rehabilitation is not physiologically feasible due to the extent of the resection, then kinematic data may still be of use as it can be used to characterize successful compensatory strategies. These may be subsequently taught to other individuals treated for O&OSCC.

Our second aim was to assess to what extent the above mentioned changes related to the clinical variables of TNM staging, tumour location, and treatment modality. Overall, our results show that individuals with smaller tumours are less affected in terms of articulatory–kinematic changes as compared with those with larger tumours. This is most likely due to the fact that, in general, smaller excisions lead to fewer problems with mobility, and a more mobile tongue is associated with better speech outcomes (Bressmann et al., 2004; Chepeha et al., 2016; Lam & Samman, 2013; van Dijk et al., 2016). Moreover, if less tissue is resected, more tissue is left to form (near) complete constrictions against the palate, resulting in reduced airflow escape from the oral cavity with a better pressure build‐up as a result.

Despite some variability, the results of most studies do indicate that movement is most impacted in the region of the tumour location in the oral or oropharyngeal cavity. That is, an anterior tumour on the tongue will likely affect movement in the anterior region of the tongue. This is most likely due to a combination of tissue loss, and scar tissue at the site of the resection, which results in atypical muscle patterns and subsequent movements. Knowing how tumour location affects the articulatory–kinematic movement following treatment is beneficial to clinicians. Information regarding expected speech outcomes may aid in informing patients and benefits shared decision‐making. Moreover, this knowledge may help in designing therapeutic interventions post‐treatment. No studies assessed the effect of primary and/or adjuvant radiation therapy on kinematic speech outcomes in individuals treated for O&SSC, which precludes us from answering this question.

The final aim of our review was to assess how articulatory–kinematics change over time in individuals treated for O&SSC. The results were highly variable in this regard as studies reported both improvements (Hamlet et al., 1990, 1992; Kansy et al., 2017, 2018) and further deterioration of speech (Acher et al., 2014) as time following surgical treatment increased. One potential reason for the conflicting results is that the number of individuals who received treatment for O&OSCC was small (five for Hamlet et al., 1990, 1992; two for Acher et al., 2014; and one for Kansy et al., 2017, 2018). This highlights the need for more relevant prospective data concerning the development of articulatory–kinematics following treatment for O&OSCC, as SLPs presently experience this lack of relevant evidence as a barrier to clinical practice (Blyth et al., 2024).

The evidence presented in this review was limited, both in terms of quality and quantity. First, not all studies provided thorough descriptions of the patient group in terms of tumour characteristics and in‐ and exclusion criteria. Second, it is very likely that a selection bias existed in terms of the included individuals who received treatment for O&OSCC. Only a few included studies mentioned inclusion criteria, and recruiting strategies were rarely mentioned. Third, many studies did not have matched control groups in terms of age, which may be problematic considering that speech motor control is affected by age (Mücke et al., 2020; Thies et al., 2022). Future work should provide a more detailed description of the included individuals, recruitment strategies, and carefully match them to control speakers. These methodological considerations would also reduce the risk of bias that was present in many studies included in our review.

Limitations in terms of quantity of the evidence concern the absence of studies assessing the effect of primary and adjuvant (chemo)radiation therapy on the articulatory–kinematics. While these forms of treatment spare the organ, articulatory–acoustic studies have already shown that radiation‐based therapies compromise articulation as well (Jacobi et al., 2016, 2013). Second, prospective studies were limited to a 12‐month follow‐up time, making it impossible to assess long‐term changes. Especially if individuals received adjuvant radiation therapy, a worsening of speech may be found in the long term as shown by an acoustic evaluation of voice quality with a 10‐year follow‐up period (Karsten et al., 2020).

There were also limitations to our review process. We only synthesized results regarding the articulatory–kinematic changes of speech following O&OSCC treatment, leaving the interrelationships between kinematic, acoustic, and perceptual changes for future work. Second, no meta‐analysis was conducted in our review due to the heterogeneity of both the group of individuals treated for O&OSCC and speech assessment methods, as well as due to the fact that many studies provided qualitative rather than quantitative descriptions of kinematic patterns.

Our review contains implications for practice and research for individuals treated for O&OSCC. The overall number of individuals treated for O&OSCC was low and only six studies included more than ten individuals treated for O&OSCC. Moreover, most studies had a heterogeneous group of patients. This highlights the critical need for a comprehensive research programme targeting the articulatory–kinematic consequences of treatment for O&OSCC in larger and more homogenous patient groups. Currently, SLPs experience a lack of relevant evidence as a barrier to clinical practice (Blyth et al., 2024). Given the increase in long‐term O&OSCC survivors and the negative impact speech impairments have on quality of life, it is crucial that this issue is addressed. Well‐designed kinematic studies with a larger sample size may bring us toward evidence‐based and standardized speech therapy, which is currently absent for individuals treated for O&OSCC (Blyth et al., 2015; Bressmann, 2021). The synthesized compensatory strategies in this review may serve as a starting point to systematically and formally assess which compensatory behaviour may provide optimal gains in speech intelligibility or acceptability. Moreover, the varying methodologies and stimuli choices show that standardized measurement tools need to be developed in order to strengthen the comparability across studies, a point already recognized in 2012 (Schuster & Stelzle, 2012). The absence of standardized measurement tools seem to be emblematic for individuals with head and neck cancer as this specific issue has been noted for individuals treated for laryngeal cancers as well (van Sluis et al., 2018).

## CONCLUSIONS

This systematic review consisted of 29 studies and assessed the articulatory–kinematic consequences of treatment for oral and oropharyngeal cancer. Due to the presence of moderate to high risk of bias in most included studies, the results of the review need to be interpreted with caution. In terms of outcomes, our review noted both global and local changes. Global changes included a stiffening of the tongue, a difficulty in controlling different parts of the tongue in tandem, and more asymmetrical movement between the affected and unaffected side. Local changes included difficulties with tongue‐tip raising and fronting, and curling back the tongue in order to make constrictions in the velar region. In general, these changes were related to tumour size and location. Smaller tumours resulted in fewer or less pronounced kinematic changes and tumour location corresponded to the place of experienced articulatory difficulty. Conflicting results were found regarding the development of kinematic changes following treatment. Our review further highlighted the critical need of assessing the articulatory–kinematic consequences of oral and oropharyngeal cancer treatment in larger, better described, and more homogenous patient populations with well‐matched control groups. This would help to inform rehabilitation strategies that need to be developed in order to meet patient needs in follow‐up care for those with O&OSCC.

## CONFLICT OF INTEREST STATEMENT

The authors report no conflict of interest

## Supporting information



Supplementary Materials

## Data Availability

Data‐sharing is not applicable to this article as no new data were created or analysed in this study.

## ENDNOTE

1 [https://www.crd.york.ac.uk/prospero/display_record.php?ID=CRD42022340489]
