# Supplementary material for: Articulatory–kinematic changes in speech following surgical treatment for oral or oropharyngeal cancer: A systematic review
Source: Int J Lang Commun Disord. 2024 Dec 18;60(1):e13148. doi: 10.1111/1460-6984.13148 (PMC11654356; doi:10.1111/1460-6984.13148)
Supplement: Supplementary file 1 — Supplementary Materials [file JLCD-60-0-s001.docx]

**Supplementary Materials**

**S-Table 1.** Full search string for each database.

| **Database** | **Search string** | **Clarification** |
| --- | --- | --- |
| PubMed | (Mouth Neoplasms[Mesh] OR Oropharyngeal Neoplasms[Mesh] OR Facial Neoplasms[Mesh] OR Head and Neck Neoplasms[Mesh] OR Tongue Neoplasms[Mesh] OR Oral squamous cell carcinoma[Title/Abstract] OR Squamous cell carcinoma[Title/Abstract] OR Oral cancer[Title/Abstract] OR  Oral tumo*[Title/Abstract] OR Oral carcinoma[Title/Abstract] OR  Mouth cancer[Title/Abstract] OR Mouth tumo*[Title/Abstract] OR  Mouth carcinoma[Title/Abstract] OR Oropharyngeal cancer[Title/Abstract] OR Oropharyngeal tumo*[Title/Abstract] OR Oropharyngeal carcinoma[Title/Abstract] OR Head and neck cancer[Title/Abstract] OR Head and neck tumo*[Title/Abstract] OR  Head and neck carcinoma[Title/Abstract] OR Facial cancer[Title/Abstract] OR Facial tumo*[Title/Abstract] OR  Facial carcinoma[Title/Abstract] OR Tongue cancer[Title/Abstract] OR Tongue tumo*[Title/Abstract] OR Tongue carcinoma[Title/Abstract] OR Glossectom*[Title/Abstract] OR  Post-glossectom*[Title/Abstract] OR Postglossectom*[Title/Abstract] ) AND (Articulation Disorders[Mesh] OR Speech Intelligibility[Mesh] OR movement[Title/Abstract] OR articulation[Title/Abstract] OR  speech[Title/Abstract] OR intelligibility[Title/Abstract] OR voice[Title/Abstract] OR acousti*[Title/Abstract] OR phoneti*[Title/Abstract] OR Speech perception[Title/Abstract] OR  Speech therapy[Title/Abstract] OR tongue displacement[Title/Abstract] OR tongue motion[Title/Abstract] OR  tongue positio*[Title/Abstract] OR Lingual displacement[Title/Abstract] OR Jaw displacement[Title/Abstract] OR lingual movement[Title/Abstract] OR Tongue movement[Title/Abstract] OR Jaw movement[Title/Abstract] OR  lip displacement[Title/Abstract] OR lip movement[Title/Abstract] OR lip aperture[Title/Abstract] OR asymmetr*[Title/Abstract] OR  symmetr*[Title/Abstract] OR concav*[Title/Abstract] OR  tongue tip elevation[Title/Abstract]) AND (magnetic resonance imag*[Title/Abstract] OR MRI[Title/Abstract] OR rt-MRI[Title/Abstract] OR rtMRI[Title/Abstract] OR Real-time MRI[Title/Abstract] OR cine-MRI[Title/Abstract] OR ultrasound[Title/Abstract] OR UTI[Title/Abstract] OR  ultrasound tongue imaging[Title/Abstract] OR EMA[Title/Abstract] OR Electromagnetic articulography[Title/Abstract] OR EPG[Title/Abstract] OR Electropalatography[Title/Abstract] OR  Palatography[Title/Abstract] OR vocal tract[Title/Abstract] OR  linguopalatal contact[Title/Abstract] OR Videofluoroscop*[Title/Abstract] OR X-ray[Title/Abstract] OR  X-ray microbeam[Title/Abstract]) | [Title/Abstract]  = limit search to  title and abstract |
| PsychInfo | AB(“Oral squamous cell carcinoma” OR “squamous cell carcinoma” OR “Oral cancer” OR “Oral tumo*” OR“Oral carcinoma” OR “Mouth cancer” OR  “Mouth tumo*” OR “Mouth carcinoma” OR “Oropharyngeal cancer” OR “Oropharyngeal tumo*” OR “Oropharyngeal carcinoma” OR “Head and neck cancer” OR “Head and neck tumo*” OR “Head and neck carcinoma” OR “Facial cancer” OR “Facial tumo*” OR “Facial carcinoma” OR “Tongue cancer” OR “Tongue tumo*” OR “Tongue carcinoma” OR Glossectom* OR Post-glossectom* OR Postglossectom*) AB(“movement” OR “articulation” OR “speech” OR “intelligibility” OR “voice” OR “acousti*” OR “phoneti*” OR “speech perception” OR “Speech therapy” OR “tongue displacement” OR “Tongue motion” OR “Tongue positio*” OR “lingual movement” OR “Lingual displacement” OR “Jaw displacement” OR “Tongue movement” OR  “Jaw movement” OR “asymmetr*” OR “symmetr*” OR “Lip displacement” OR “Lip movement” OR “Lip aperture” OR “concav*” OR "tongue tip elevation") AB(“magnetic resonance imag*” OR “MRI” OR “rt-MRI” OR "rtMRI" OR “Real-time MRI” OR “cine-MRI” OR “ultrasound” OR “UTI” OR ”ultrasound tongue imaging” OR “EMA” OR “Electromagnetic articulography” OR “EPG” OR “Electropalatography” OR “Palatography” OR “vocal tract” OR “linguopalatal contact” OR “Videofluoroscop*” OR “X-ray” OR “X-ray microbeam”) | AB =  limit search to  abstract |
| Scopus | TITLE-ABS-KEY(“Oral squamous cell carcinoma” OR “squamous cell carcinoma” OR “Oral cancer” OR “Oral tumo*” OR “Oral carcinoma” OR “Mouth cancer” OR “Mouth tumo*” OR “Mouth carcinoma” OR “Oropharyngeal cancer” OR “Oropharyngeal tumo*” OR “Oropharyngeal carcinoma” OR “Head and neck cancer” OR “Head and neck tumo*” OR “Head and neck carcinoma” OR “Facial cancer” OR “Facial tumo*” OR “Facial carcinoma” OR “Tongue cancer” OR “Tongue tumo*” OR “Tongue carcinoma” OR Glossectom* OR Post-glossectom* OR Postglossectom*) TITLE-ABS-KEY(“movement” OR “articulation” OR “speech” OR “intelligibility” OR “voice” OR “acousti*” OR “phoneti*” OR “speech perception” OR “Speech therapy” OR “tongue displacement” OR “Tongue motion” OR “Tongue positio*” OR “lingual movement” OR “Lingual displacement” OR “Jaw displacement” OR “Tongue movement” OR “Jaw movement” OR “asymmetr*” OR “symmetr*” OR “Lip displacement” OR “Lip movement” OR “Lip aperture” OR “concav*” OR "tongue tip elevation") TITLE-ABS-KEY(“magnetic resonance imag*” OR “MRI” OR “rt-MRI” OR "rtMRI" OR “Real-time MRI” OR “cine-MRI” OR “ultrasound” OR “UTI” OR ”ultrasound tongue imaging” OR “EMA” OR “Electromagnetic articulography” OR “EPG” OR “Electropalatography” OR “Palatography” OR “vocal tract” OR “linguopalatal contact” OR “Videofluoroscop*” OR “X-ray” OR “X-ray microbeam”) | TITLE-ABS-KEY =  limit search to  title, abstract and keywords |
| Web of Science | ((TS= (“Oral squamous cell carcinoma” OR “squamous cell carcinoma” OR “Oral cancer” OR “Oral tumo*” OR “Oral carcinoma” OR “Mouth cancer” OR “Mouth tumo*” OR “Mouth carcinoma” OR “Oropharyngeal cancer” OR “Oropharyngeal tumo*” OR “Oropharyngeal carcinoma” OR “Head and neck cancer” OR “Head and neck tumo*” OR “Head and neck carcinoma” OR “Facial cancer” OR “Facial tumo*” OR “Facial carcinoma” OR “Tongue cancer” OR “Tongue tumo*” OR “Tongue carcinoma” OR Glossectom* OR Post-glossectom* OR Postglossectom*)) AND TS=(“movement” OR “articulation” OR “speech” OR “intelligibility” OR “voice” OR “acousti*” OR “phoneti*” OR “speech perception” OR “Speech therapy” OR “tongue displacement” OR “Tongue motion” OR “Tongue positio*” OR “lingual movement” OR “Lingual displacement” OR “Jaw displacement” OR “Tongue movement” OR “Jaw movement” OR “asymmetr*” OR “symmetr*” OR “Lip displacement” OR “Lip movement” OR “Lip aperture” OR “concav*” OR "tongue tip elevation")) AND TS=(“magnetic resonance imag*” OR “MRI” OR “rt-MRI” OR "rtMRI" OR “Real-time MRI” OR “cine-MRI” OR “ultrasound” OR “UTI” OR ”ultrasound tongue imaging” OR “EMA” OR “Electromagnetic articulography” OR “EPG” OR “Electropalatography” OR “Palatography” OR “vocal tract” OR “linguopalatal contact” OR “Videofluoroscop*” OR “X-ray” OR “X-ray microbeam”) | TS = abstract, title, and keywords |
| Embase | “mouth tumor”/de OR “oropharynx tumor”/de OR “face tumor”/de OR “tongue tumor”/de OR “head and neck tumor”/de OR “Oral squamous cell carcinoma”:ti,ab,kw OR “squamous cell carcinoma”:ti,ab,kw OR “Oral cancer”:ti,ab,kw OR “Oral tumo*”:ti,ab,kw OR “Oral carcinoma”:ti,ab,kw OR “Mouth cancer”:ti,ab,kw OR “Mouth tumo*”:ti,ab,kw OR “Mouth carcinoma”:ti,ab,kw OR “Oropharyngeal cancer”:ti,ab,kw OR “Oropharyngeal tumo*”:ti,ab,kw OR “Oropharyngeal carcinoma”:ti,ab,kw OR “Head and neck cancer”:ti,ab,kw OR “Head and neck tumo*”:ti,ab,kw OR “Head and neck carcinoma”:ti,ab,kw OR “Facial cancer”:ti,ab,kw OR “Facial tumo*”:ti,ab,kw OR “Facial carcinoma”:ti,ab,kw OR “Tongue cancer”:ti,ab,kw OR “Tongue tumo*”:ti,ab,kw OR “Tongue carcinoma”:ti,ab,kw OR Glossectom*:ti,ab,kw OR Post-glossectom*:ti,ab,kw or Postglossectom*:ti,ab,kw AND “speech disorder”/de OR “speech intelligibility”/de OR movement:ti,ab,kw or articulat*:ti,ab,kw or speech:ti,ab,kw or intelligibility:ti,ab,kw or voice:ti,ab,kw or acousti*:ti,ab,kw or phoneti*:ti,ab,kw or “speech perception”:ti,ab,kw or “Speech therapy”:ti,ab,kw or “tongue displacement“:ti,ab,kw or “Tongue motion”:ti,ab,kw or “Tongue positio*”:ti,ab,kw or “lingual movement”:ti,ab,kw or “Lingual displacement”:ti,ab,kw or “Jaw displacement”:ti,ab,kw or “Tongue movement”:ti,ab,kw or “Jaw movement”:ti,ab,kw or “Lip displacement”:ti,ab,kw or “Lip movement”:ti,ab,kw or “Lip aperture”:ti,ab,kw or asymmetr*:ti,ab,kw or symmetr*:ti,ab,kw or concav*:ti,ab,kw or "tongue tip elevation":ti,ab,kw AND “magnetic resonance imag*”:ti,ab,kw or MRI:ti,ab,kw or “rt-MRI”:ti,ab,kw or rtMRI:ti,ab,kw or “Real-time MRI”:ti,ab,kw or “cine-MRI“:ti,ab,kw or ultrasound:ti,ab,kw or UTI:ti,ab,kw or “ultrasound tongue imaging”:ti,ab,kw or EMA:ti,ab,kw or “Electromagnetic articulography”:ti,ab,kw or EPG:ti,ab,kw or Electropalatography:ti,ab,kw or Palatography:ti,ab,kw or “vocal tract”:ti,ab,kw or “linguopalatal contact”:ti,ab,kw or Videofluoroscop*:ti,ab,kw or X-ray:ti,ab,kw or “X-ray microbeam”:ti,ab,kw | ti,ab,kw = limit search to title, abstract, and author keywords  de = index term |
